# Metabolic Unhealthiness Increases the Likelihood of Having Metabolic Syndrome Components in Normoweight Young Adults

**DOI:** 10.3390/ijerph16183258

**Published:** 2019-09-05

**Authors:** Bagas Suryo Bintoro, Yen-Chun Fan, Chia-Chi Chou, Kuo-Liong Chien, Chyi-Huey Bai

**Affiliations:** 1 International Master/Ph.D. Program in Medicine, College of Medicine, Taipei Medical University, Taipei 11031, Taiwan; 2 Department of Health Behavior, Environment, and Social Medicine, Faculty of Medicine, Public Health, and Nursing, Universitas Gadjah Mada, Yogyakarta 55281, Indonesia; 3 School of Public Health, College of Public Health, Taipei Medical University, Taipei 11031, Taiwan; 4 Department of Internal Medicine, Keelung Chang Gung Memorial Hospital, Keelung 20401, Taiwan; 5 Institute of Preventive Medicine, College of Public Health, National Taiwan University, Taipei 11031, Taiwan; 6 Department of Internal Medicine, National Taiwan University Hospital, Taipei 10002, Taiwan; 7 Department of Public Health, College of Medicine, Taipei Medical University, Taipei 11031, Taiwan

**Keywords:** metabolic syndrome, insulin resistance, cardiovascular disease prevention, metabolic health, national survey, cohort, young adult

## Abstract

Findings on risk detection for having metabolic syndrome (MetS) components, each of which may individually increase the risk of disease and mortality, are limited in young adults. In this study, we aimed to calculate the likelihood of having ≥1 MetS component in normoweight young adults using two different metabolic health criteria. We recruited 1182 normoweight young adults from the Taiwan Survey on the Prevalence of Hypertension, Hyperglycemia, and Hyperlipidemia and the National Health Interview Survey (aged 16–45 years, 39% male, body mass index = 18.5–22.99, all without MetS) and followed them for 5 years. Metabolic health criteria were derived from the Harmonized criteria (unhealthy if showing abnormality in one or two MetS components) and the triglyceride-glucose index (TyG-i; unhealthy if TyG-i was in the >75th percentile). Odds ratio (OR) and 95% confidence interval (CI) estimations for having ≥1 MetS component and for having each MetS component in 5 years were assessed using multivariable-adjusted logistic regression. We observed a significantly increased likelihood of the presence of ≥1 MetS component in the unhealthy group by using the Harmonized criteria and TyG-i (adjusted OR (aOR); 95%CI: 2.64; 2.02–3.45 and 2.1; 1.57–2.82, respectively). The areas under the receiver-operating characteristics curves were 0.679 and 0.652 for the final models using Harmonized and TyG-i criteria, respectively. These findings support the recommendation of treating any metabolic component abnormality, even in young adults without a MetS diagnosis.

## 1. Introduction

Maintaining metabolic measurements within the ideal range (e.g., ideal body weight and lipid profile) is critical to reduce the incident of cardiovascular disease (CVD) risk [1]. Metabolic syndrome (MetS) is a metabolic disorder cluster, which can be a risk factor for CVD and type 2 diabetes mellitus (T2DM), with insulin resistance as the hallmark. MetS prevalence is increasing, particularly in children and adolescents [2,3]. Of the many MetS diagnostic criteria, the Harmonized Criteria state that a diagnosis should be made in the presence of ≥3 of 5 components—namely elevated blood pressure (BP), elevated triglyceride (TG) level, elevated fasting plasma glucose (FPG) level, elevated waist circumference (WC), and reduced high-density lipoprotein cholesterol (HDL-C) level [4].

MetS diagnosis is the most widely used criterion to define metabolic health status [5,6,7,8]. Studies have combined body mass index (BMI) and metabolic health status to predict diseases such as hypertension [6], CVD [9], and T2DM [10]. TG–glucose index (TyG-i), the product of FPG and TG levels, has been found to be a possible insulin resistance marker in recent years [11,12,13]. TyG-i performance research for distinguishing metabolic health is also increasing [14,15], along with the investigation of its disease prediction performance for hypertension [16], T2DM [17,18], and major adverse cardiovascular events (MACEs) [19]. TyG-i requires only two simple laboratory parameters, which can be measured easily and economically [13].

In addition to MetS diagnosis, every MetS component can increase the all-cause and cardiovascular mortality risk [20]. The presence of 1 or 2 MetS components is also described as stage B in the MetS evolution, and medical treatment is recommended [21]. Individuals without MetS diagnosis but with one or two MetS components would possibly be categorized as healthy, and there may be a late detection. Thus, early identification of people with MetS risk is essential [22]. Moreover, in young adults, MetS prevalence is only 4.8–7%; however, one third of them have at least one MetS component [23]. Thus, compared with MetS diagnosis, detecting the presence of MetS components may be more essential.

Relevant studies on young adults remain limited, particularly in the Chinese population. Young adults are more likely to be in good health. The low absolute risk, calculated using the Systematic COronary Risk Evaluation system for CVD, may reveal a very high relative risk in young adults with a high level of risk factor, which requires intensive lifestyle advice [24]. Most rapid weight gain occurs at the age of 20–40 years [2], stressing the need for understanding metabolic health beyond the progressive body weight in the young population. Young adults have been neglected and have been assessed less for CVD risk [25].

The purpose of this study was to calculate the likelihood of having any MetS component, as defined by Harmonized Criteria [4], in normoweight young adults. Harmonized Criteria were refined from the National Cholesterol Education Program Adult Treatment Panel III definition by grading all the five components as equal, but suggesting a region-specific value for the WC cutoff [4]. This approach was selected as the most appropriate for our study to elucidate the metabolic health dynamic with less interference of BMI. We compared two metabolic health criteria for risk prediction, namely a scoring system based on Harmonized Criteria and TyG-i. Another study objective was to determine the prediction performance of both metabolic health criteria.

## 2. Materials and Methods

### 2.1. Study Population

**Figure 1:**
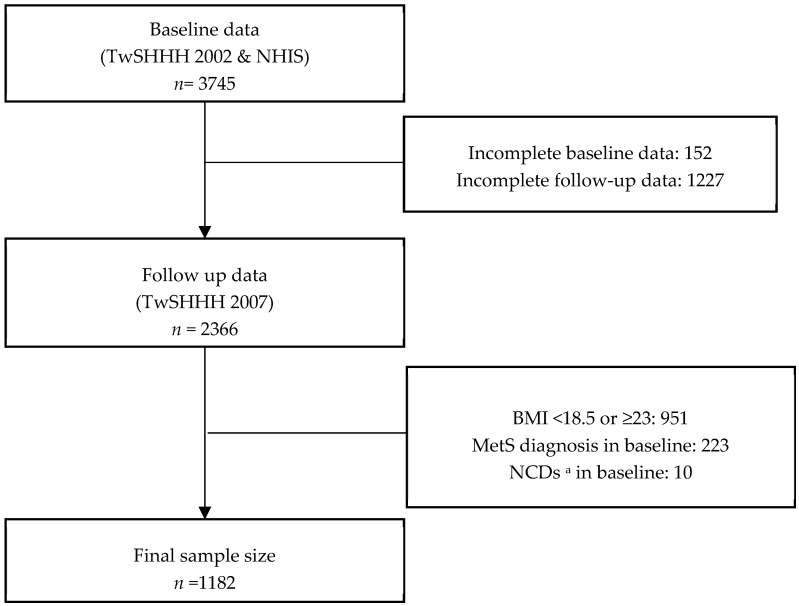
Sample selection process from 2001 National Health Interview Survey (NHIS) and 2002 and 2007 Taiwan Survey on the Prevalence of Hypertension, Hyperglycemia, and Hyperlipidemia (TwSHHH) respondents. Abbreviations: *n*, number of respondents; MetS, metabolic syndrome. ^a^ Selected noncommunicable disease (i.e., hypertension, dyslipidemia, and type 2 diabetes mellitus (T2DM)).

This cohort study was conducted using two datasets: the Taiwan Survey on the Prevalence of Hypertension, Hyperglycemia, and Hyperlipidemia (TwSHHH) and the National Health Interview Survey (NHIS). The NHIS was conducted in 2001 using multistage stratified systematic sampling. The first TwSHHH was conducted in 2002 on NHIS participants. The combination of these two datasets served as our baseline data. The follow-up data were derived from the second TwSHHH in 2007. The inclusion criteria were BMI = 18.5–22.99 kg/m^2^ (normoweight) and age of 16–45 years. The BMI cutoff used was described as a public health action point in Asia [26]. The exclusion criteria were: prevalent MetS diagnosis as per Harmonized Criteria or selected noncommunicable diseases (NCDs; i.e., T2DM, hypertension, and/or hyperlipidemia at baseline, as diagnosed by a health professional and followed by medical treatment); erroneous and incomplete laboratory values; and loss to follow-up. From 3745 individuals aged 16–45 years, the final sample size selected was 1182 respondents (Figure 1). The TwSHHH was approved by the Institutional Review Board and Ethics Committee of the Bureau of Health Promotion, Department of Health (Taipei, Taiwan; approval number: N201704074). All TwSHHH and NHIS respondents signed an informed consent document before data collection.

### 2.2. MetS

A trained nurse performed the anthropometric measurements under the TwSHHH standardized protocol. BMI was calculated based on measured height and weight (in kg/m^2^). WC was measured to the closest centimeter. The detailed procedure, including biomarker sampling and analysis, was described previously [27]. MetS components were defined using Harmonized Criteria: (1) elevated WC (≥90 cm for Asian men or ≥80 cm for Asian women), (2) elevated TG level (≥150 mg/dL or receiving drug treatment), (3) reduced HDL-C level (≤40 mg/dL for men or ≤50 mg/dL for women, or receiving drug treatment), (4) elevated systolic/diastolic BP (≥130/85 mmHg or receiving drug treatment), and (5) elevated FPG level (≥100 mg/dL or receiving drug treatment) [4]. We included a person who reported taking hyperlipidemia drugs into both the elevated TG and reduced HDL-C groups.

In our study, we defined an individual as metabolically unhealthy if the respondent showed abnormality in 1 or 2 MetS components, according to Harmonized Criteria. The term unhealthy-Har was used in this study for consistency. The TyG-i was calculated as:

Natural logarithm (Ln) [fasting triglycerides (mg/dL) × fasting glucose (mg/dL)/2 [28].

In the TyG-i scoring system, metabolically unhealthy (unhealthy-TyG) was defined as a respondent with TyG-i ≥ 75th percentile (Q4).

### 2.3. Outcome

We determined the primary outcome as the presence of ≥1 MetS component in the follow-up. The secondary outcome was the presence of each MetS component in the follow-up.

### 2.4. Covariates

Age was categorized into three groups: 16–25, >25–35, and >35–45. For alcohol consumption, the products of alcohol frequency and alcohol amount were then categorized into low and high consumption and nonconsumption. Respondents reported positive current alcohol consumption; however, respondents reporting no data on alcohol frequency or consumption amount were categorized under “low-alcohol consumption” (*n* = 4). Fatty food consumption was assessed using the question, “How often do you eat fatty foods (e.g., fried vegetables, animal fat/skin, poultry fat/skin, fried meat/chicken, yolk, fried tofu, fried bean product/tofu, fried fish, lard, and oily sauce)?” The responses were rated on a 5-point scale and then summed up and divided into two categories by the median, namely, low and high fried-food consumption. Vegetable and fruit consumption were coded as daily and nondaily consumption of fruit and vegetables. Family disease history was based on the self-reported history of selected NCDs, namely T2DM, hypertension, heart disease, stroke, and dyslipidemia, in parents and siblings. The positive family disease history of each NCD was summed. Binary categories for smoking behavior (nonsmoker or smoker) and exercise status (routine exercise or no exercise) were applied. Total cholesterol (TC) level, baseline BMI, family disease history, and age were used as continuous covariates.

### 2.5. Statistical Analysis

Statistical analyses were performed using STATA software (version 14, StataCorp LLC: College Station, TX, USA). Sample characteristics are presented as proportions based on the metabolic health category. We used Student’s *t* test (or Wilcoxon rank-sum test for continuous variables) and χ^2^ (or Fisher exact test for dichotomous measures) to test for differences in baseline and follow-up characteristics between the groups.

We employed logistic regression by using the healthy category as the reference to calculate the odds ratios (ORs) and 95% confidence intervals (95% CIs) of having the outcomes. First, we calculated crude ORs and then developed a model adjusted for sex, age, baseline BMI, TC level, alcohol consumption, fatty food consumption, vegetable and fruit consumption, smoking habit, exercise, and family disease history. To determine the effect of sex and age, we performed sex and age-stratified analysis. To resolve our second research question, we calculated the area under the curve (AUC) of the receiver-operating characteristics (ROC) curve to compare the adjusted model using Harmonized Criteria and TyG-i with the main outcome (i.e., the risk of metabolic unhealthiness in 5 years defined as having ≥1 MetS component). To assess whether the model could improve our prediction, we also compared these criteria to baseline MetS components for the outcome predictions.

## 3. Results

### 3.1. Baseline and Follow-Up Characteristics

Our study sample comprised 1182 individuals, 468 (39.6%) of whom were male. Table 1 lists the age, lifestyle, family disease history, and biochemical measures of our sample population. No significant differences in alcohol, vegetable, fruit, fatty food consumption, or exercise were observed between the healthy and unhealthy groups based on Harmonized Criteria or the TyG-i. Both criteria presented significant differences in parent disease history. TyG-i also demonstrated significant differences in sex, smoking habit, sibling disease history, and age. In terms of biomarkers, both criteria demonstrated significant differences in MetS components between the metabolically healthy and unhealthy groups.

### 3.2. Baseline Prevalence, Follow-Up Prevalence, Incidence, and Remission Rate for MetS Components

Approximately 30% of the study population had at least one MetS component at baseline; this value increased to 40% by 2007. A reduced HDL-C level was the most prevalent MetS component in 2002 (17.9%) and 2007 (21.2%). In the healthy individuals, the incidence and remission of metabolic unhealthiness, defined by having abnormal values in 1 or 2 MetS components, were 327 and 418 per 1000 people, respectively (Table 2). A reduced HDL level was the MetS component with the highest incidence (166 per 1000 people), whereas an elevated FPG level was the MetS component with the highest remission rate (829 per 1000 people).

### 3.3. Risk of Having ≥1 MetS Component

Table 3 presents the ORs of having ≥1 MetS component and of having each MetS component in metabolically unhealthy individuals based on both criteria (Harmonized Criteria and TyG-i). Compared with that observed for metabolically healthy individuals, the crude OR (cOR) for having ≥1 MetS component within 5 years increased for individuals classified as metabolically unhealthy based on Harmonized Criteria (cOR = 2.87, 95% CI = 2.22–3.71). Increased likelihood of having ≥1 MetS component was also noted if the individual was classified as metabolically unhealthy based on TyG-i (cOR = 2.32, 95% CI = 1.77–3.03; Table 3). The likelihood of having each MetS component also increased if individual was classified as unhealthy, with comparable values being observed between the two criteria.

In a multivariate model, we noted a significant increase in the likelihood of having ≥1 MetS component without much difference in the adjusted ORs (aORs) of Harmonized Criteria (aOR = 2.64, 95% CI = 2.02–3.45) and TyG-i (aOR = 2.11, 95% CI = 1.57–2.82). When an individual exhibited metabolic unhealthiness, the likelihood of having an elevated TG level (Harmonized Criteria [aOR = 3.01, 95% CI = 1.96 –4.61] and TyG-i [aOR = 5.64, 95% CI = 3.58–8.87]) and a reduced HDL level (Harmonized Criteria [aOR = 2.53, 95% CI = 1.85 –3.46] and TyG-i [aOR = 2.63, 95% CI = 1.85–3.74]) within 5-years increased significantly. Being unhealthy as per the TyG-i definition did not significantly increase the likelihood of having an elevated WC (aOR = 1.59, 95% CI = 0.99–2.56) or the likelihood of having an elevated BP (aOR = 1.45, 95% CI = 0.96–2.19). Both criteria indicated no significant increase in the likelihood of having an elevated FPG level in the adjusted models (Table 3).

In the sex- and age-stratified analyses, we used a multivariate-adjusted model to better understand the potential effect of sex and age group on the relationship between being metabolically unhealthy and the likelihood of possessing ≥1 MetS component (Table A1). Being metabolically unhealthy, as defined by Harmonized Criteria or TyG-i, increased the risk of having ≥1 MetS component and each MetS components in sex- and age-stratified analyses.

### 3.4. ROC Curves for the Risk of Having ≥1 MetS Component

**Figure 2:**
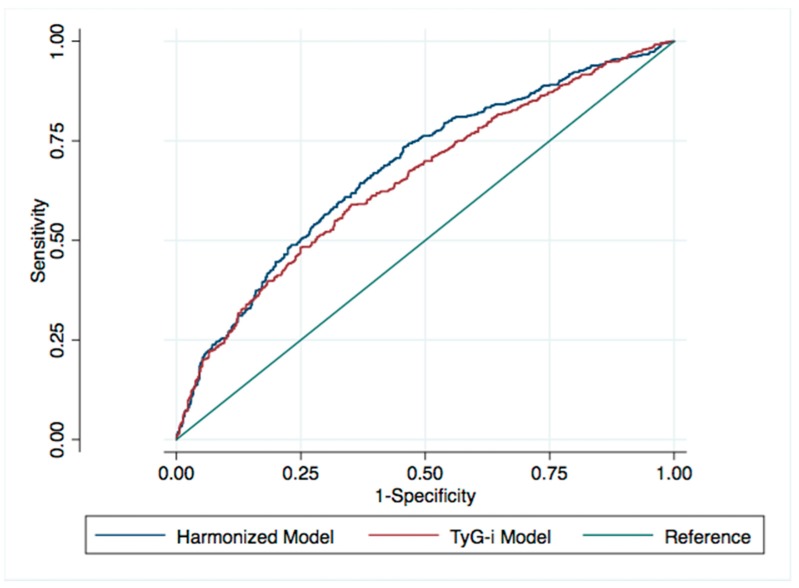
ROC curve comparison for final models in normoweight young adults. Both models are adjusted for sex, age, baseline BMI, TC, alcohol consumption, fatty food consumption, vegetable and fruit consumption, smoking habit, exercise, and family disease history.

For the Harmonized Criteria and TyG-i, the AUCs were 0.679 (95% CI = 0.648–0.710) and 0.652 (95% CI = 0.620–0.684), respectively, for the final model used to define metabolic health (Figure 2). A prediction model with an AUC value of 0.5 corresponds to random prediction. Higher AUC value indicates better prediction performance and the maximum AUC value is 1.0 [29]. The Harmonized Criteria exhibited significantly better results than did TyG-i in predicting the main outcome. They also demonstrated superior values to all components, whereas TyG-i was superior to only the elevated baseline FPG levels and elevated WC (Table 4).

## 4. Discussion

In our normoweight young adult population study, being metabolically unhealthy, defined as either having ≥1 MetS component of the Harmonized Criteria or being in the top quartile of TyG-i, increased the likelihood of having ≥1 MetS component in 5 years of follow-up for both definitions, across the sex and age groups. We employed a stricter definition for metabolically unhealthy as being component-free, instead of diagnosis-free, per the Harmonized Criteria. Compared with the Harmonized Criteria-based model, the TyG-i-based model demonstrated lower prediction power.

Our study stressed that the findings were relevant to the youngest age group of 16–45 years, independent of several known risk factors for MetS components, such as age, TC level, BMI, lifestyle, and family disease history. Our results warrant that in this study, the presence of metabolic components drives the increase in the likelihood of having MetS components, rather than BMI. Moreover, this association is less likely to be driven by extreme values because in this study, we excluded individuals who had prevalent MetS diagnosis and NCDs.

The absence of a significant association between metabolic health status and having high WC in 5 years follow-up in age- and sex-stratified analyses was likely due to the association of BMI with WC. Moreover, follow-up WC was more related to baseline BMI and baseline WC (beta-coefficient = 2.72, 95% CI = 2.41–3.03 and beta-coefficient = 0.69, 95% CI = 0.64–0.74, respectively; all in continuous scale), indicating that higher BMI and WC increases the risk of having higher WC, despite implementing a stricter cutoff (because in Asian populations, BMI ≥ 23 is considered overweight). The wide probability range of ORs (i.e., 95% CI) in the likelihood of elevated FPG might be explained by the fact that in this population, the prevalences of this condition were low both at baseline and at follow-up. In Taiwan, T2DM incidence in 20–59-year-old individuals is less than half compared to that in 60–79-year-old individuals, for both sexes [30]. Elevated FPG levels are the least prevalent component of MetS in young adults [31]; their prevalence being even lower than that of the MetS diagnosis [23]. The insulin secretion begins to increase during the early insulin resistance development through β-cell compensation, such that FPG level is maintained at the normal level. The decompensated condition during exhaustion may trigger the elevation of FPG levels [32]. In our target population, young adults were most likely to maintain normal glucose metabolism during the compensated period, both at baseline and at the 5-year follow-up.

The Harmonized Criteria-based model performed significantly better than that based on TyG-i and also significantly better than each baseline MetS criterion. Simplifying the prediction by placing the individuals having 1 or 2 MetS components in the same group did not significantly affect the model performance (Table A2). This showed that the superiority of the model was not driven by a person having two components in the model as an extreme value. Although MetS is related to insulin resistance and TyG-i was shown to be highly predictive of current [12] and future [33] insulin resistance, the association of TyG-i with the study outcome was not stronger than that of MetS components. Martínez et al. [17] concluded that the TyG-i was significantly associated with a higher T2DM risk in a Caucasian adult population (mean age = 55 years). Although the maximum AUC in the current study was 0.679, it was the better than that of TyG-i or individual MetS components. Studies on MetS diagnosis prediction in the overweight individual have reported that prediction does not improve by combining all the MetS components compared to the combination of three components prediction [34]. Predicting MetS components remains challenging, particularly in young healthy people as represented by the non-MetS normoweight population in this study.

In Taiwan, MetS prevalence is approximately 15%; with age its prevalence increases—from 5.2% in those aged 20–29 years to 36.5% in those aged 70–79 years [27]. Moreover, it is higher among Taiwan metropolitan adults [35]. The risk of having MetS components in young adults is critical because they are soon to become middle-aged. In a study, middle-aged men with MetS diagnosis had increased risks of cardiovascular events and total death, regardless of their BMI status, over >30 years of follow-up [36]. If not overall MetS diagnosis, every individual MetS component can increase CVD risk [20]. The detrimental effect increases as the number of these components increases [20,37]. MetS also increases MACE risk and all-cause mortality in patients who undergo revascularization [38]. MetS progression status can also increase the risk of dementia [39].

Our finding supports the recommendation of administering treatment for MetS component abnormality, even if the person has not received a MetS diagnosis [21]. Thus, every health examination result in this age period, such as college admission- or job recruitment-related health check-ups, should be addressed appropriately. Health providers should be informed that no metabolic measurements should be underestimated, irrespective of age. Moreover, this study found that the remission of MetS components in 5 years following the usual care strategy is considerably high. Research analyzing remission in the absence of intervention remains rare [40]. Treating the risk factors should be beneficial because dietary intervention and usual care could lead to MetS remission in >50% of patients with MetS [40]. Because this study included only normoweight young adults, the dynamic interaction between their metabolic health and their MetS status throughout the individuals’ lifecycles requires a wider study because CVD is preventable [41] and every prevention effort is crucial [42].

To the best of our knowledge, this is the first study to investigate the risk of having MetS components in non-MetS normoweight Asian young adults. This study employed a representative sample of the Taiwanese population in 2002 and thus may represent well the normoweight population dynamic in Taiwan; this is because the young adult group, dominated by nonoverweight individuals [43], has increased, coinciding with the global trend [2]. We performed biomarker and anthropometric measurements to classify participants into specific metabolic health status categories. Previous studies have included unhealthy populations and/or demonstrated only the risk of mortality. Moreover, they have included participants with wider age distributions, not analyzed the effect on the age-stratified group, and not focused on young adults [19,44]. The inclusion of stricter metabolic health definitions and younger individuals in our study should aid in providing further insight into the treatment for the MetS-related preclinical condition. Predicting the treatable preclinical condition is essential to reduce the risk through many evidence-based interventions within a sufficient timespan. Risk prediction for objectively measurable outcomes would also make it easier for the health provider to implement risk communication strategies.

This study has several limitations. This study followed respondents for 5 years after the first TwSHHH in 2002; however, the actual length of the metabolic heath condition remained unknown. Moreover, we accommodated medication as a diagnosis criterion, and thus we did not control for medication use during the follow-up. Nevertheless, by excluding individuals taking medication at baseline and by using medication as an outcome criterion, the related interference could have been diminished. In Taiwan, young adults are less likely to take regular medication, such as that for hypertension (3% of 19–44-year old men and 8% of 19–44-year old women) [45]. Our study focused on young adults with normal weight and no MetS diagnosis. The generalizability of our findings may thus be limited, and our results might not be applicable to the general population. Finally, our study outcomes were inclusive for incident and prevalent cases. By using this approach, we demonstrated that the presence of a metabolic abnormality may increase the likelihood of having the same condition in a 5-year timespan.

## 5. Conclusions

Being metabolically unhealthy, based on either the Harmonized Criteria or TyG-i, is an independent risk factor for MetS component possession in non-MetS normoweight young adults. The MetS components provide adequate, relevant information about the adverse health effects of the condition, even with a current normal BMI; this information may aid patient management. Our findings support the recommendation of treating any metabolic component abnormality, even in young adults without a MetS diagnosis.

## Figures and Tables

**Table 1:** Baseline and follow-up characteristics.

Variables	Harmonized Criteria					TyG-Index					Total (*n* = 1182)	
	Healthy (*n* = 835) (0 MetS Component)		Unhealthy (*n* = 347) (1–2 MetS Component)		*p*-Value	Healthy (*n* = 887) (TyG-i ≤ 8.41)		Unhealthy (*n* = 295) (TyG-i > 8.41)		*p*-Value		
	%		%			%		%			%	
Baseline												
Male	39.8		39.2		0.85	37.2		46.8		0.004	39.6	
Smoking (yes)	15.9		19.0		0.19	15.2		21.7		0.01	16.8	
Alcohol (no)	81.9		77.2		0.14	81.6		77.3		0.102	80.5	
Vegetable & Fruit (daily)	86.2		82.4		0.09	84.8		86.1		0.581	85.1	
Fatty food (low)	56.9		56.9		1.00	54.7		51.5		0.347	53.9	
Exercise (routine)	78.5		79.5		0.71	78.7		79.3		0.819	78.9	
PDH (yes)	44.1		53.6		0.003	44.4		54.2		0.003	53.1	
SDH (yes)	9.0		12.4		0.075	8.5		14.6		0.002	10.0	
	Mean	SD	Mean	SD		Mean	SD	Mean	SD		Mean	SD
Age	30.1	8.4	30.8	8.8	0.19	29.5	8.5	32.8	8.0	<0.001	30.3	8.5
BMI	20.7	1.2	21.0	1.2	<0.001	20.6	1.2	21.2	1.2	<0.001	20.8	1.2
TG ^a^	76.0	34.0	95.0	32.0	<0.001	71.0	27.0	130.0	42.0	<0.001	90.0	46.1
SBP	103.9	9.7	107.6	13.7	<0.001	104.0	10.9	108.0	11.4	<0.001	104.9	11.1
DBP	67.9	7.7	72.1	10.2	<0.001	68.4	8.5	71.5	8.7	<0.001	69.1	8.7
WC	71.1	6.1	73.7	7.5	<0.001	71.0	6.3	74.4	7.1	<0.001	71.8	6.6
HDL	60.4	11.0	47.6	12.1	<0.001	57.5	12.2	54.2	14.2	<0.001	56.6	12.7
FPG	84.1	6.6	88.2	10.8	<0.001	84.4	7.6	88.2	9.4	<0.001	85.3	8.2
Follow-up												
BMI	21.8	2.4	22.3	2.4	0.001	21.7	2.4	22.5	2.4	<0.001	21.9	2.4
TG ^a^	77.0	42.0	90.0	70.0	<0.001	72.0	37.0	110.0	69.0	<0.001	94.2	55.2
SBP	109.9	11.5	113.6	15.0	<0.001	110.0	12.2	114.0	13.6	<0.001	110.8	12.7
DBP	70.4	8.5	73.7	11.1	<0.001	70.5	9.0	73.9	10.2	<0.001	71.2	9.4
WC	74.6	7.3	76.3	7.9	<0.001	74.3	7.2	77.5	7.9	<0.001	75.1	7.5
HDL	55.8	10.7	50.3	9.5	<0.001	55.3	10.8	51.0	9.9	<0.001	54.3	10.8
FPG	82.4	7.5	85.2	10.1	<0.001	82.2	7.4	86.3	10.4	<0.001	83.1	8.4

PDH, parent disease history; SDH, sibling disease history; TG, triglyceride; TyG-index, TG–glucose index; SBP, systolic blood pressure; DBP, diastolic blood pressure; WC, waist circumference; HDL, high-density lipoprotein; FPG, fasting plasma glucose; SD, standard deviation. ^a^ Median and interquartile ranges (Wilcoxon rank-sum test).

**Table 2:** Baseline prevalence, follow-up prevalence, incidence rate, and remission rate of MetS components, based on Harmonized Criteria.

		Outcome					
		≥1 MetS	Elevated TG	Elevated BP	Elevated WC	Reduced HDL	Elevated FPG
Baseline	n/N	347/1182	87/1182	55/1182	21/1182	211/1182	41/1182
	%	29.4	7.4	4.7	1.8	17.9	3.5
Follow-up	n/N	475/1182	120/1182	142/1182	105/1182	250/1182	28/1182
	%	40.2	10.2	12.0	8.9	21.2	2.4
Incidence	per 1000	327	76	103	82	166	18
Remission	per 1000	418	575	527	524	578	829

MetS, metabolic syndrome; TG, triglyceride; TyG, TG–glucose index; BP, blood pressure; WC, waist circumference; HDL, high-density lipoprotein; FPG, fasting plasma glucose; *n*, number of diseased individuals; N, number of individuals at risk.

**Table 3:** Odd ratios of outcomes according to the Harmonized Criteria and TyG-i (with healthy individuals as the reference in each criterion).

	Outcome					
	≥1 MetS	Elevated TG	Elevated BP	Elevated WC	Reduced HDL	Elevated FPG
	cOR (95%CI)	cOR (95%CI)	cOR (95%CI)	cOR (95%CI)	cOR (95%CI)	cOR (95%CI)
Unhealthy-Har	2.87	3.03	2.27	1.84	2.68	2.47
	(2.22–3.71) ^z^	(2.06–4.44) ^z^	(1.59–3.24) ^z^	(1.22–2.78) ^y^	(2.01–3.59) ^z^	(1.16–5.23) ^x^
Unhealthy-TyG	2.32	7.88	2.11	1.98	1.73	3.11
	(1.77–3.03) ^z^	(5.24–11.85) ^z^	(1.46–3.04) ^z^	(1.31–3.01) ^y^	(1.28–2.34) ^z^	(1.46–6.60) ^y^
	aOR (95%CI)	aOR (95%CI)	aOR (95%CI)	aOR (95%CI)	aOR (95%CI)	aOR (95%CI)
Unhealthy-Har	2.64	3.01	1.99	1.56	2.53	2.21
	(2.02-3.45) ^z^	(1.96-4.61) ^z^	(1.35-2.94) ^z^	(1.01-2.42) ^x^	(1.85-3.46) ^z^	(0.99-4.91)
Unhealthy-TyG	2.11	5.64	1.45	1.59	2.63	1.93
	(1.57-2.82) ^z^	(3.58-8.87) ^z^	(0.96-2.19)	(0.99-2.56)	(1.85-3.74) ^z^	(0.83-4.48)

Abbreviations, same as in Table 2. Unhealthy-Har: metabolically unhealthy based on Harmonized Criteria, defined as a respondent having abnormal values in 1 or 2 MetS components according to Harmonized Criteria; Unhealthy-TyG: metabolically unhealthy based on TyG-i criteria, defined as a respondent having TyG-i ≥ 75th percentile (Q4). cOR, crude odds ratio; aOR, adjusted odds ratio. Data adjusted for sex, age, baseline BMI, TC, alcohol consumption, fatty food consumption, vegetable and fruit consumption, smoking habit, exercise, and family disease history. ^x^ *p* < 0.05, ^y^ *p* < 0.01, ^z^ *p* < 0.001.

**Table 4:** AUC comparison of models for 5-year risk of having ≥1 MetS component.

Model	AU-ROC	Lower 95%CI	Upper 95%CI
Harmonized	0.679	0.647	0.710
TyG-Index ^y^	0.652	0.619	0.684
Elevated FPG ^y,z^	0.631	0.598	0.663
Elevated BP ^y^	0.659	0.627	0.690
Elevated TG ^y^	0.651	0.619	0.683
Elevated WC ^y,z^	0.631	0.599	0.663
Low HDL ^y^	0.639	0.607	0.672

^y^ *p* < 0.05 compared to Harmonized Criteria, ^z^ *p* < 0.05 compared to TyG-i criterion. All models are adjusted for sex, age, baseline BMI, TC, alcohol consumption, fatty food consumption, vegetable and fruit consumption, smoking habit, exercise, and family disease history. Abbreviations, same as in Table 2.

## Appendix A

**Table A1:** OR for outcome of having components, according to the Harmonized Criteria and the TyG index, in normoweight young adults (with healthy individuals as the reference in each criterion).

		aOR	95%CI	aOR	95%CI	aOR	95%CI
**Sex Stratification**							
		**≥1 MetS**		**Elevated TG**		**Elevated BP**	
Female	Harmonized	2.78	(1.96–3.95) ^z^	3.33	(1.60–6.94) ^y^	3.8	(1.93–7.49) ^z^
	TyG-I	2.06	(1.40–3.05) ^z^	9.97	(4.36–22.80) ^z^	1.21	(0.59–2.48)
Male	Harmonized	2.29	(1.47–3.57) ^z^	2.95	(1.72–5.05) ^z^	1.56	(0.95–2.58)
	TyG-I	2.24	(1.42–3.53) ^z^	4.53	(2.58–7.94) ^z^	1.62	(0.96–2.73)
		**Elevated WC**		**Reduced HDL**		**Elevated FPG**	
Female	Harmonized	1.75	(1.01–3.04) ^x^	2.53	(1.75–3.65) ^z^	2.93	(1.00–8.55) ^x^
	TyG-I	1.38	(0.75–2.53)	2.88	(1.89–4.40) ^z^	2.24	(0.75–6.70)
Male	Harmonized	1.37	(0.63–2.96)	2.41	(1.28–4.56) ^y^	1.86	(0.51–6.80)
	TyG-I	2.03	(0.91–4.53)	2.31	(1.18–4.54) ^x^	1.64	(0.44–6.12)
**Age Stratification**							
		**≥1 MetS**		**Elevated TG**		**Elevated BP**	
15–25	Harmonized	2.77	(1.72–4.46) ^z^	4.92	(1.88–12.92) ^y^	2	(0.95–4.18)
	TyG-I	1.87	(1.04–3.38) ^x^	6.14	(2.31–16.33) ^z^	2.81	(1.25–6.30) ^x^
>25–35	Harmonized	2.5	(1.50–4.16) ^z^	4.24	(1.95–9.22) ^z^	1.3	(0.54–3.12)
	TyG-I	2.97	(1.75–5.03) ^z^	6.28	(2.81–14.03) ^z^	2.16	(0.91–5.13)
>35–45	Harmonized	2.73	(1.74–4.27) ^z^	2.05	(1.02–4.12) ^x^	2.69	(1.48–4.91) ^y^
	TyG-I	1.71	(1.09–2.69) ^x^	5.39	(2.54–11.43) ^z^	0.86	(0.46–1.63)
		**Elevated WC**		**Reduced HDL**		**Elevated FPG**	
15–25	Harmonized	1.65	(0.78–3.48)	2.53	(1.44–4.47) ^y^	-	-
	TyG-I	1.73	(0.72–4.16)	2.29	(1.08–4.83) ^x^	-	-
>25–35	Harmonized	1.68	(0.65–4.35)	2.77	(1.57–4.89) ^z^	1.68	(0.38–7.45)
	TyG-I	1.34	(0.51–3.50)	3.28	(1.80–5.96) ^z^	1.33	(0.33–5.37)
>35–45	Harmonized	1.58	(0.76–3.26)	2.87	(1.65–4.97) ^z^	3.88	(1.16–13.03) ^x^
	TyG-I	1.72	(0.80–3.66)	2.64	(1.47–4.74) ^y^	3.17	(0.89–11.31)

aOR, adjusted odd ratio, adjusted for sex, age, baseline body mass index (BMI), total cholesterol (TC), alcohol consumption, fatty food consumption, vegetable and fruit consumption, smoking habit, exercise, and family disease history. MetS, metabolic syndrome; TG, triglyceride; TyG-i, TG–glucose index; BP, blood pressure; WC, waist circumference; HDL, high-density lipoprotein; FPG, fasting plasma glucose. ^x^ *p* < 0.05, ^y^ *p* < 0.01, ^z^ *p* < 0.001.

**Table A2:** OR of outcome according to the number of components based on the Harmonized Criteria in normoweight young adults.

	Outcome					
	≥1 MetS	Elevated TG	Elevated BP	Elevated WC	Reduced HDL	Elevated FPG
MetS comp.	cOR 95%CI	cOR 95%CI	cOR 95%CI	cOR 95%CI	cOR 95%CI	cOR 95%CI
1 Comp.	2.69	2.56	1.89	1.59	2.51	1.07
	(2.04–3.55) ^z^	(1.68–3.89) ^z^	(1.27–2.81) ^y^	(1.01–2.50) ^x^	(1.84–3.43) ^z^	(0.38–3.00)
2 Comp.	3.77	5.29	4.1	3	3.48	8.95
	(2.25–6.34) ^z^	(2.92–9.59) ^z^	(2.32–7.27) ^z^	(1.55–5.79) ^y^	(2.07–5.85) ^z^	(3.72–21.53) ^z^
	aOR 95%CI	aOR 95%CI	aOR 95%CI	aOR 95%CI	aOR 95%CI	aOR 95%CI
1 Comp.	2.56	2.69	1.78	1.41	2.28	0.97
	(1.92–3.42) ^z^	(1.69–4.28) ^z^	(1.16–2.72) ^y^	(0.87–2.27)	(1.63–3.19) ^z^	(0.33–2.87)
2 Comp.	3.01	4.28	2.89	2.2	3.93	7.73
	(1.77–5.14) ^z^	(2.17–8.45) ^z^	(1.54–5.42) ^z^	(1.08–4.46) ^x^	(2.23–6.92) ^z^	(2.96–20.19) ^z^

Abbreviations, same as in Table A1. Comp., component. Adjusted for sex, age, baseline BMI, TC, alcohol consumption, fatty food consumption, vegetable and fruit consumption, smoking habit, exercise, and family disease history. ^x^ *p* < 0.05, ^y^ *p* < 0.01, ^z^ *p* < 0.001.
